# Z-Score Linear Discriminant Analysis for EEG Based Brain-Computer Interfaces

**DOI:** 10.1371/journal.pone.0074433

**Published:** 2013-09-13

**Authors:** Rui Zhang, Peng Xu, Lanjin Guo, Yangsong Zhang, Peiyang Li, Dezhong Yao

**Affiliations:** Key Laboratory for NeuroInformation of Ministry of Education, School of Life Science and Technology, University of Electronic Science and Technology of China, Chengdu, China; University of Texas School of Public Health, United States of America

## Abstract

Linear discriminant analysis (LDA) is one of the most popular classification algorithms for brain-computer interfaces (BCI). LDA assumes Gaussian distribution of the data, with equal covariance matrices for the concerned classes, however, the assumption is not usually held in actual BCI applications, where the heteroscedastic class distributions are usually observed. This paper proposes an enhanced version of LDA, namely z-score linear discriminant analysis (Z-LDA), which introduces a new decision boundary definition strategy to handle with the heteroscedastic class distributions. Z-LDA defines decision boundary through z-score utilizing both mean and standard deviation information of the projected data, which can adaptively adjust the decision boundary to fit for heteroscedastic distribution situation. Results derived from both simulation dataset and two actual BCI datasets consistently show that Z-LDA achieves significantly higher average classification accuracies than conventional LDA, indicating the superiority of the new proposed decision boundary definition strategy.

## Introduction

Brain-computer interfaces (BCI) provide direct connection channel between brain and external world without any peripheral muscular activity [1]. It translates brain activity to signals that control external devices, and there are many augmentative communication and control systems based on BCI [2]–[6], which improves lives of people with severe neuromuscular disorders.

Generally an EEG based BCI consists of four modules [1]: 1) signal acquisition module to record and amplify EEG signals; 2) feature extraction module to extract signal features that encode user’s intent; 3) translation module to translate features into device command; 4) feedback and control module to synchronize user’s action and achieve control of external devices. The high performance EEG amplifier with suitable reference strategy [7] will increase quality of the recorded EEG signal, and employing innovative paradigms in the feedback and control module may obtain higher quality features and better control strategies [8]–[11]. Once EEG amplifier as well as reference strategy, and the feedback and control module are determined, feature extraction and translation algorithms will play important roles in improving the performance of BCI. Currently, the conventional features used in scalp EEG based BCI can be attributed to event related potentials, the sensorimotor rhythm, the transient visual potentials and the steady state potentials (including visual and audio). To refine those specific features, many feature extraction algorithms have been proposed [12]–[15]. However, as an input-output system, the final translation module directly determines whether the subject’s intention is correctly decoded [16]. Compared to the conventional pattern recognition problems, BCI system requires the translation module to have ability to handle with the small sample size training problem, the heteroscedastic class distribution problem and the nonstationary physiological signals, etc. Therefore, effective translation algorithms specifically suitable for BCI application are still required in BCI discipline [17] [18].

Linear discriminant analysis (LDA) is one of the most popular classification algorithms for BCI application, and has been successfully used in a great number of BCI systems such as motor imagery based BCI [19], P300 speller [20] and steady state movement related potentials based BCI [21]. The original LDA has two derivations [22], fisher LDA (FLDA) and least square LDA (LSLDA). FLDA is based on Fisher-Rao’s criterion [22]–[24], which is to find the projection 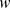 to maximize the objective function 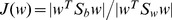 denoting the ratio of between-class to within-class variances. LSLDA is derived from a linear discriminant function 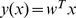, where the weight vector 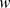 is supposed to minimize the mean squared error between 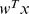 and 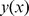 [25]. The solution of LSLDA will be equivalent to that of FLDA with a proper label coding scheme adopted in LSLDA [25].

Both the two kind of LDAs are under the homoscedasticity assumption that different classes follow Gaussian distribution with same covariance matrices. However, the EEG data recorded from actual BCI system usually have heteroscedastic class distributions, which violates the fundamental assumption of LDA and notably degrades the recognition performance. Heteroscedastic LDA (HLDA) is an extension of FLDA, whose between-class scatter is generalized from Euclidean distance to Chernoff distance with both effects of the class means and their covariance matrices considered [26], thus HLDA does not need the homoscedasticity assumption. Nonparametric discriminant analysis (NDA) is another extension of FLDA [27], which makes no prior assumption for the class distributions, the parameters can be estimated by k nearest neighbor method and then they are used to define the between-class scatter [28].

In essence, LDA linearly transforms data from high dimensional space to low dimensional space, and finally the decision is made in the low dimensional space, thus the definition of the decision boundary plays an important role on the recognition performance. Conventional LDA defines the mean of the projected data as the decision boundary due to the homoscedasticity assumption [25]. Nearest neighbor of classes has also been proposed to serve as the decision boundary [29]. Different from LDA, support vector machine (SVM) firstly maps the data to a high dimensional space, and then finds a hyperplane in the high dimensional space so that the distance from the hyperplane to the nearest data point on each side is maximized [30], theoretically the hyperplane is determined only by a small amount of the training data which are called support vectors. During the classification procedure of LDA, the heteroscedastic class distributions will be still kept in the projected space. Therefore, we argue that if the mean and variance of the projected data could be considered for the definition of the decision boundary, it may extend LDA to deal with the practical heteroscedastic distribution data, which is the derivation point for the proposed Z-LDA in this paper.

The paper is organized as follows. Section *Methods and Materials* provides a detailed description of z-score LDA (Z-LDA); The results of simulation dataset and motor imagery EEG datasets are showed in section *Results*; In section *Discussions*, there is a general discussion of the proposed algorithm; Section *Conclusion* gives a summary of this work.

## Materials and Methods

### 1. Linear Discriminant Analysis

To simplify the description of the algorithm, we only consider the case of two classes. Assume 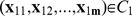 and 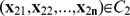, with 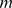 and 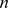 being the number of samples, are the samples in the two class sets 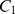 and 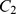. Let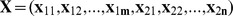, then the simplest representation of a linear discriminant function is obtained by taking a linear function of the input vector so that
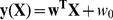
(1)where 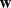 is called a weight vector, and 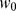 is a bias. Using vector notation, equation (1) can be converted to
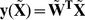
(2)where 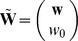 and 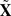 is the corresponding augmented input vector 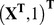 with a dummy input 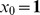. Accordingly, the least square solution of equation (2) is [25]



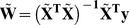
(3)With 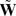 estimated in equation (3), the corresponding weight sum 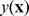 can be achieved. For conventional LDA, classification for an input 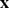 is based on the comparison of 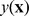 and threshold, i.e., the decision boundary. If we consider 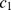 as the label of class 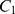,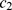 as the label of class 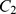, the corresponding decision boundary can be defined by 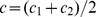.

### 2. Z-score Linear Discriminant Analysis

Theoretically, the decision boundary of LDA is derived by assuming the homoscedasticity distribution for the two classes. Thus it may not be competitive to the heteroscedastic distribution, and we will develop the following strategy to define a more robust decision boundary.

Based on the estimated 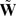 obtained by equation (3), the weight sum 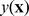 for each training sample can be calculated from equation (2), and then the parameters of the Gaussian distributions of the weight sum 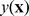 related to the two classes can be estimated as,
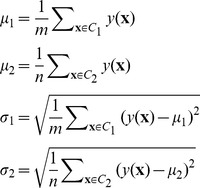
(4)where 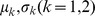 are the corresponding mean and standard deviation (SD) of the weight sum 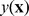 for training set 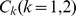. During classification, when a new sample 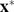 is input, firstly calculate the weight sum 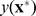 by equation (2), then perform the following normalization procedure,



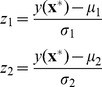
(5)In essence, 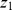 and 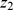 are the transformed z-scores to measure how much the weight sum 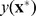 of the newly input sample is close to the two weight sum distributions predefined by the training set, thus the method is called z-score linear discriminant analysis (Z-LDA). Finally, if 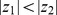, the sample is classified into 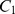, otherwise, the sample belongs to 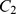. Assume the weight sum of samples in the two classes subject to Gaussian distribution with parameters 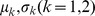, then the proposed decision boundary is the intersection of the two Gaussian distribution curves.

The above descriptions are based on LSLDA, since the only difference between LSLDA and FLDA is the way to estimate the weight vector 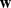, and the solutions of them are substantially equal. Therefore, the proposed decision boundary definition strategy can be extended to FLDA, too.

### 3. Relationship between LDA and Z-LDA

Theoretically, the decision boundary of conventional LDA is defined by
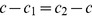
(6)


Based on equation (6), the decision boundary of conventional LDA is the mean of labels of two classes, i.e. 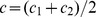. Obviously, when the SDs are combined into classification, the decision boundary of Z-LDA is defined as
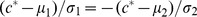
(7)which deduces a value



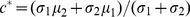
(8)Apparently, the decision boundary 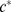 of Z-LDA is defined by both the mean and SD of the weight sum of two classes.

In the binary classification, the expectation of mean of the weight sum 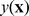 for training set is 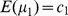
and 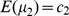, the decision boundary of conventional LDA is theoretically equal to 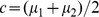. When the weight sum of two classes have equal SDs, the decision boundary of Z-LDA will also reduce to 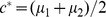. Therefore, the conventional LDA is a particular case of Z-LDA.

## Results

### 1. Evaluation on Simulation Dataset

#### 1.1. Simulation dataset description

In this section, we constructed a simulated dataset in order to investigate the capability of Z-LDA in dealing with the two class classification with heteroscedastic class distributions. The simulation was performed by using the fundamental two 2-dimensional Gaussian distributions, where the samples in the first class follow a Gaussian distribution with mean (−*1,* −*0.6*) and standard deviation (*0.3, 0.3*), and the samples in the second class follow a Gaussian distribution with mean (*1, 0.6*) and standard deviation (*0.3, 0.3*). To generate datasets with heteroscedastic class distributions, we changed the SD of the second class step by step, and then performed the comparison between LDA and Z-LDA on those datasets. Training set and test set with each consisting of 200 2-dimensional samples (100 for each class) were generated respectively.

#### 1.2. Classification performance of LDA and Z-LDA

**Figure 1:**
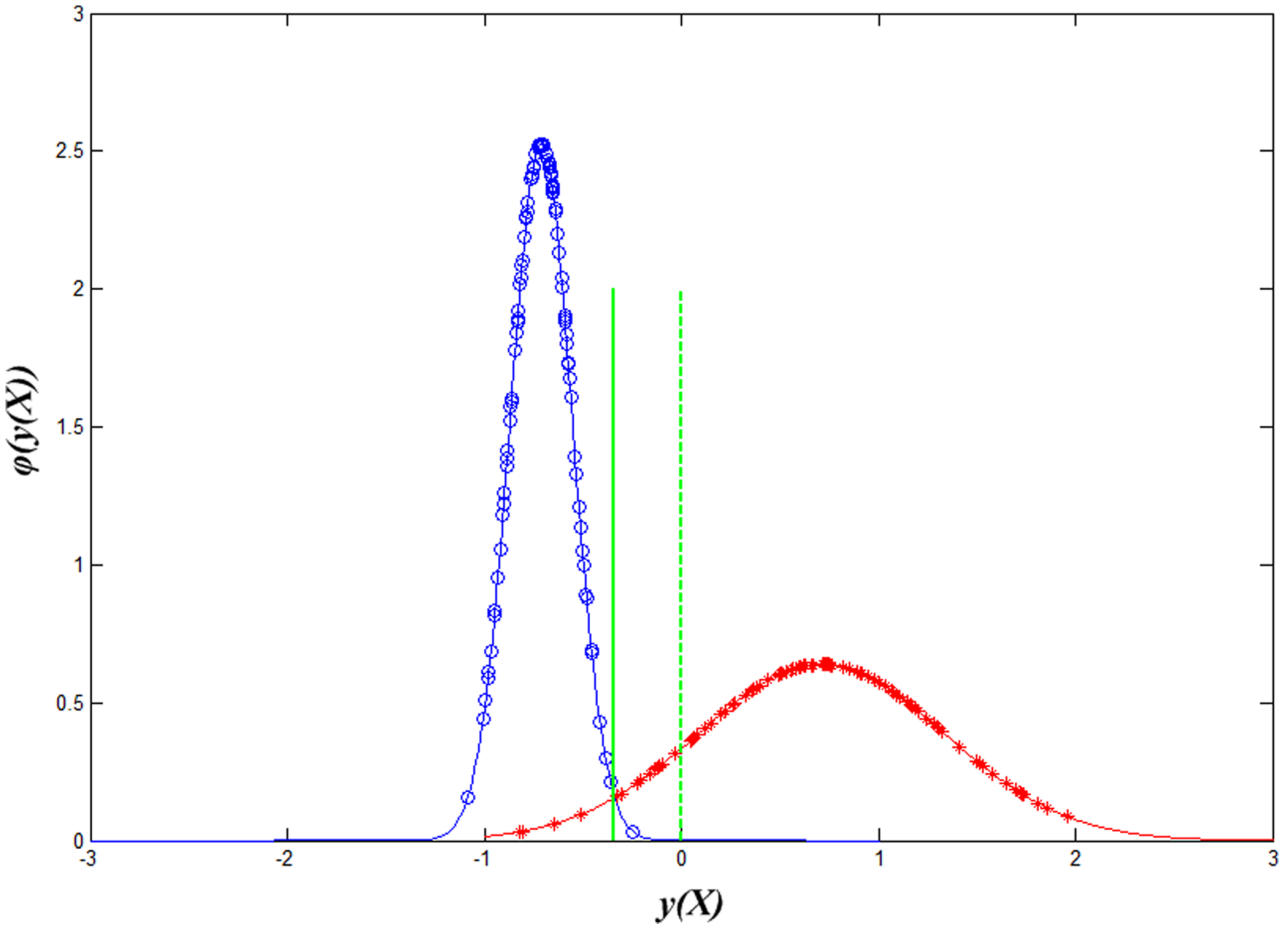
The decision boundaries of Z-LDA and LDA defined from training set. Blue circles are the weight sum 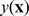 of the first class, blue solid line denotes the Gaussian distribution curve they subject to; red stars are the weight sum 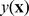 of the second class, red solid line denotes the Gaussian distribution curve they subject to; green dashed line denotes the decision boundary of LDA, 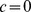, and green solid line denotes the decision boundary of Z-LDA, 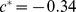.

**Figure 2:**
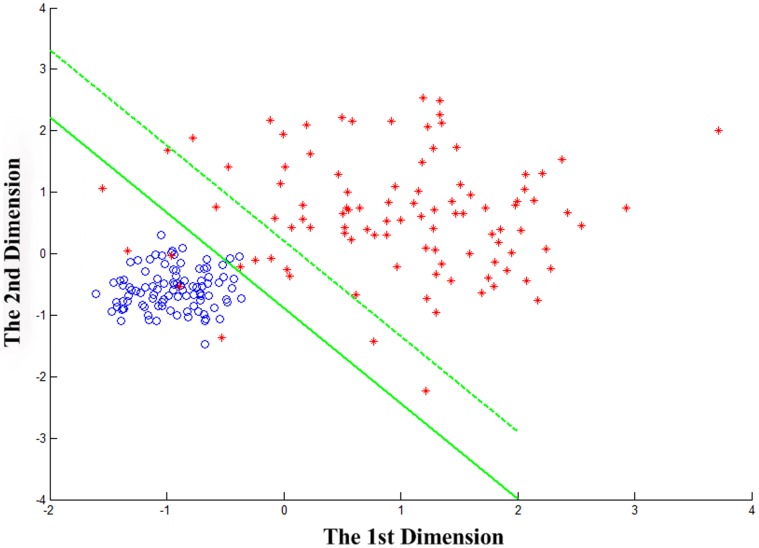
The classification performance of Z-LDA and LDA on test samples. Blue circles are the test samples of the first class; red stars are the test samples of the second class; green dashed line denotes the decision boundary of LDA; green solid line denotes the decision boundary of Z-LDA.

After the simulated datasets with heteroscedastic class distributions are generated, the training model of LDA and Z-LDA were estimated from the training set respectively, and the models were then applied to classify the samples in the test set. The above procedure was repeated 100 times to lower the random effect, and paired t-test was performed to investigate whether the statistical difference exists between the two classifiers. Table 1 listed the mean and standard deviation of classification accuracies for the 100 runs. Figure 1 visually gived the decision boundary definition procedure for the two classifiers when the standard deviation of the first class is *(0.3, 0.3)*, and *(1.0, 1.0)* for the second class. Figure 2 intuitively showed the difference of recognition performance for the test dataset based on the two decision boundaries in Figure 1.

**Table 1:** Classification accuracies (%) of Z-LDA and LDA on simulation dataset.

Difference of SDs^a^	0	0.1r^b^	0.2r	0.3r	0.4r	0.5r	0.6r	0.7r	0.8r	0.9r
**LDA**	**99.99±0.05**	99.91±0.22	99.51±0.49	98.79±0.77	97.56±1.09	96.27±1.39	95.15±1.39	93.98±1.38	92.70±1.94	91.70±1.72
**Z-LDA**	**99.99±0.05**	**99.97±0.13**	**99.78±0.35**	**99.39±0.63**	**98.95±0.72**	**98.16±0.97**	**97.09±1.18**	**96.22±1.37**	**95.01±1.53**	**93.85±2.19**
**p-value**		0.0153	<10^−5^	<10^−8^	<10^−20^	<10^−22^	<10^−20^	<10^−23^	<10^−17^	<10^−12^

a denotes the norm of the difference of SDs between the two simulated dataset.

b r is a constant value, equals to 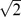.

When SDs of two classes are same, LDA and Z-LDA achieved equal classification accuracy. But while we changed the SD of the second class with that of the first class kept, though the classification accuracies for both two classifiers are lowered, Z-LDA achieved higher classification accuracy than LDA. Paired t-test revealed that when the difference of SD between the two classes exists, Z-LDA would achieve the significantly higher classification accuracy than that of LDA (p<0.05), where the more obvious improvement could be observed for those simulations with more differences in SD.

### 2. Evaluations on Real BCI Dataset

#### 2.1. BCI dataset description

##### 1) Dataset IVa of BCI Competition III

This dataset contains EEG signals recorded from five subjects using 118 electrodes [31]. In each trial, a visual cue was shown for 3.5 s, during which three motor imageries were performed, i.e., left hand, right hand and right foot. The motor imageries of right hand and foot were needed to be classified. The total number of EEG trials for each subject was 280. Specifically, 168, 224, 84, 56, and 28 trials were used as training data corresponding to the five subjects: *aa*, *al*, *av*, *aw*, and *ay*, respectively. The data were band-pass filtered between 0.05 and 200 Hz and down-sampled at 100 Hz for succedent analysis.

##### 2) Dataset recorded by our BCI system

This dataset comes from our group, consists of EEG data from 14 subjects (11 males and 3 females, right handed, 19–25 years old). The experimental protocol was approved by the Institution Research Ethics Board at University of Electronic Science & Technology of China. All participants were asked to read and sign an informed consent form before participating in the study. After experiment, all the participants received a monetary compensation for their time and effort. Subjects sat in a comfortable armchair in front of a computer screen, they were asked to perform motor imagery with left hand or right hand according to the instructions appeared on the screen. Motor imagery lasts for 5 seconds, and follows a 5 seconds rest. 15 Ag/AgCl electrodes covers sensorimotor area were used for EEG recordings with Symtop Amplifier (Symtop Instrument, Beijing, China), the signals were sampled with 1000 Hz and band pass filtered between 0.5 Hz and 45 Hz. 4 runs on the same day were recorded for each subject, each run consists of 50 trials, 25 trials for each class, and there is a 3 minutes break between the consecutive two runs. The first 2 runs are treated as training set and the last 2 runs are treated as test set.

#### 2.2. Preprocessing

We used the EEG segments recorded from 0.5 s to 3.75 s after the visual cue for the following analysis according to [32] on the first dataset. For the second dataset, all the EEG segments during motor imagery were selected for analysis, and those trials with absolute amplitude above 300 µv threshold were considered to be contaminated with strong ocular artifacts and will be removed from analysis. Next, the specific optimal frequency band for each subject was obtained by 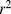 [33], and then it was used to design band pass filter for the selected EEG segments.

#### 2.3. Feature extraction

Common spatial pattern (CSP) analysis was used to estimate the spatial projection matrix, which projects the EEG signal from original sensor space to a surrogate sensor space [13], [19]. Each row vector of the projection matrix is a spatial filter, which maximizes the variance of the spatially filtered signal under one task while minimizing the variance of the spatially filtered signal under the other task. The most discriminative 3 pairs of optimal spatial filters in the projection matrix were selected to transform the band pass filtered EEG signal, then the logarithm of the variance of the transformed surrogate channel EEG signals were served as the final features for task recognition. In general, each EEG segment was transformed to a 6-dimensional vector feature after the above procedure.

#### 2.4. Classification results

In this section, we will compare the classification performance of Z-LDA to LDA, SVM, NDA and HLDA. LIBSVM with default parameter was served as SVM classifier [34]. NDA in reference [28] with 5 as the number of k nearest neighbors, and HLDA in reference [26] were used for evaluation in current work.

The classification results of Dataset IVa of BCI Competition III were summarized in Table 2. Z-LDA and NDA achieved higher average accuracy than LDA, SVM and HLDA. Though the average accuracy of NDA is slightly larger than that of Z-LDA, Z-LDA had the better performance for 4 of 5 subjects with exception for subject *ay*, and the paired t test did not show the statistical difference between them (p = 0.4146). There are only 28 training samples for subject *ay*, which is a small size training problem. NDA is good at dealing with the small size training problem, resulting in the obvious improvement for subject *ay*. Across the 5 subjects, when LDA is regarded as the baseline for evaluation, only Z-LDA showed the consistent improvement for all the 5 subjects, and the paired t test also revealed that only the accuracies obtained by Z-LDA is significantly higher than that of LDA (p = 0.0293).

**Table 2:** Classification accuracies (%) comparison on Dataset IVa of BCI Competition III.

Subjects	aa	al	av	aw	ay	Mean±Std
**LDA**	76.8	**100**	67.3	97.8	56.7	79.7±18.9
**Z-LDA**	**77.7**	**100**	68.4	**99.6**	59.9	81.1±18.2
**SVM**	75.9	**100**	**71.9**	98.2	53.6	79.9±19.4
**NDA**	75.9	**100**	64.3	97.8	70.6	81.7±16.2
**HLDA**	57.1	**100**	46.9	97.3	**72.2**	74.7±23.7

The classification results of Dataset recorded by our BCI system were summarized in Table 3. Z-LDA achieved the highest mean accuracy among the tested 5 classifiers. Paired t test also showed that the accuracies obtained by Z-LDA is significantly higher than LDA (p = 0.0004), NDA (p = 0.0006) and HLDA (p<10^−5^), but no statistical difference between Z-LDA and SVM (p = 0.0654).

**Table 3:** Classification accuracies (%) comparison on Dataset recorded by our BCI system.

Subjects	1	2	3	4	5	6	7	8	9	10	11	12	13	14	Mean±Std
**LDA**	94	84	95	69	82	63	78	**87**	70	56	**84**	81	63	**99**	78.9±13.1
**Z-LDA**	**95**	**85**	95	**71**	83	**66**	**80**	**87**	73	**59**	**84**	82	65	**99**	80.3±12.1
**SVM**	94	77	93	69	**84**	61	71	**87**	**75**	**59**	**84**	**83**	**67**	**99**	78.8±12.4
**NDA**	92	81	**96**	62	82	63	70	**87**	67	55	81	81	59	98	76.7±14.1
**HLDA**	51	79	90	51	49	47	48	49	42	48	63	43	55	55	55.0±13.7

The overall mean accuracies obtained by Z-LDA are 1.4% higher than that of LDA on both of the two BCI datasets. As shown in Table 2 and 3, we could also see that the accuracies obtained by Z-LDA is consistently better than (or at least equal to) LDA in all of the subjects.

**Figure 3:**
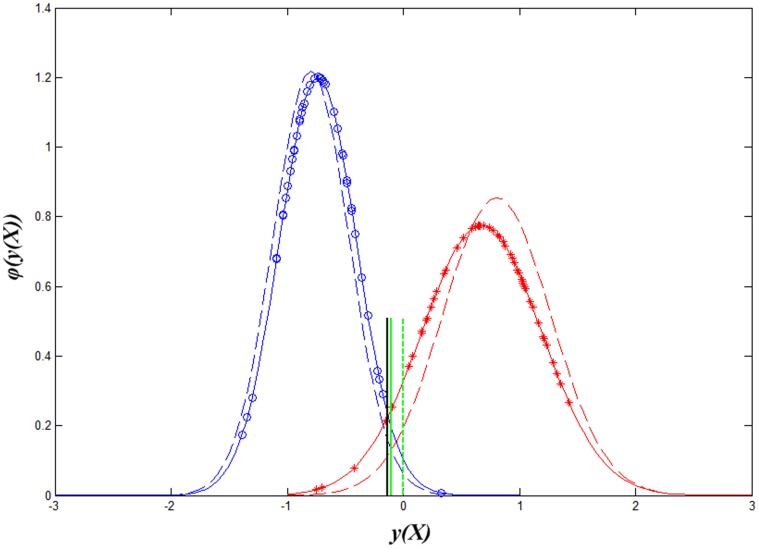
Distribution of the weight sum 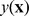 of subject 1. Blue dashed line is the Gaussian distribution curve according to the characteristic of weight sum 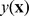 with left hand motor imagery in training set; red dashed line is the Gaussian distribution curve according to the characteristic of weight sum 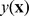 with right hand motor imagery in training set; blue circles denote the weight sum 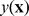 with left hand motor imagery in test set; red stars denote the weight sum 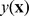 with right hand motor imagery in test set; blue solid line is the Gaussian distribution curve derived from the blue circles; red solid line is the Gaussian distribution curve derived from the red stars; green dashed vertical line is the decision boundary defined by LDA from training set; green solid vertical line is the decision boundary defined by Z-LDA from training set; black solid vertical line is the theoretical boundary of test set.

Subject 1 from Dataset recorded by our BCI system was used as an example to briefly reveal why the classification performance of Z-LDA becomes better than conventional LDA in the actual situation. The distribution of weight sum 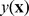 when subject 1 performed the motor imagery with left hand and right hand were plotted in Figure 3 for the training and test sets, respectively. The decision boundaries of Z-LDA and LDA were also marked in Figure 3.

## Discussion

Translation module of BCI receives features from previous feature extraction module and translates them to device command by using certain classification algorithms. In practical BCI situations, the concerned tasks may have heteroscedastic class distributions. Therefore, the consideration of effect of distribution variances may provide more robust ability to recognize the tasks. However, the conventional LDA assumes homoscedastic class distributions, which may not be competitive to handle with actual BCI dataset with heteroscedastic class distributions. Inspired by this, we develop Z-LDA by including the variance information in the classification procedure in order to provide more robust classification for BCI tasks.

As shown in Section *Methods and Materials*, the decision boundary of conventional LDA is decided by the labels of classes, while the decision boundary of Z-LDA is defined by both mean and SD of the weight sum, which is more potential to capture the distribution information of classes and provide better classification performance for heteroscedastic distribution situation.

The difference of decision boundaries between the two classifiers can be clearly observed in Figure 1. Assume we define the label of the two classes as −1 and 1, the decision boundary of LDA is fixed as 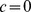, while the decision boundary of Z-LDA is determined by equation (8). If the SD of two classes are same, the decision boundary of Z-LDA is also 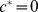, but when the SDs of two classes are different, the decision boundary of Z-LDA will move toward the class with smaller SD. From Figure 1 we can find that because of the small SD of the first class, the SD of weight sum 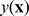 is also small, resulting in the more concentrated distribution compared to the relatively divergent distribution of class 2 with larger SD. Considering the areas under the two Gaussian curves between the two decision boundaries, the area corresponding to the second class is obviously large than that of that of the first class, which denotes that with the new defined decision boundary, more samples can be correctly recognized. Figure 2 further reveals that if the decision boundary of LDA is used in the test dataset, many samples belong to the second class will be incorrectly assigned to the first class. But if we use the decision boundary of Z-LDA to classify the samples, the number of samples which incorrectly assigned to the first class will be reduced at the cost that some samples belong to the first class will be incorrectly assigned to the second class.

When applied to the actual BCI datasets, Z-LDA consistently shows the best average accuracies among the concerned five classifiers as shown in Table 2 and Table 3. Figure 3 clearly shows us that the weight sum for the two types of tasks actually follow different Gaussian distributions in practical BCI application. In this case, the decision boundary of Z-LDA obtained from the training set is the green solid line, which is smaller than 0, and the decision boundary of LDA is the green dashed line, which equals to 0. The black solid line in Figure 3 denotes the theoretical boundary for the test dataset. Obviously, the decision boundary of Z-LDA determined by the training set is more close to the theoretical boundary of the test dataset, leading to the better classification achieved by Z-LDA compared to LDA. Therefore, we can conclude that the proposed decision boundary definition strategy outperforms the conventional decision boundary definition strategy in actual BCI applications, where concerned samples usually have the heteroscedastic distribution.

Another concerned aspect is the algorithm complexity for the online BCI system. In current work, the algorithm is implemented with Matlab R2011b running on Windows 7 Ultimate SP1 64 bit with Intel Core i5-3470 CPU 3.2 Ghz. The mean time for 200 2-dimensional samples in the simulation study using Z-LDA is 0.0004 s, and 0.0001 s for LDA. It indicates Z-LDA is applicable in the practical real time BCI.

## Conclusion

Both the simulation and actual BCI datasets confirm that Z-LDA is a more robust classification method. In essence, Z-LDA is an enhanced version of LDA, and it can be reduced to the conventional LDA by assuming homoscedastic class distributions. Moreover, the probability indicates how reliable the classification is performed could be derived from the z-score transformed weight sum, which may be helpful to handle with the adaptive calibration problem [17], [33], [35].

There are various algorithms have been proposed based on LDA in BCI application, such as regularized LDA [36], [37], Bayesian LDA (BLDA) [38] and enhanced BLDA [33]. Unlike Z-LDA, these algorithms improved LDA’s performance from other aspects like regularization, Bayesian frameworks. It is possible to combine the proposed decision boundary definition strategy with these algorithms, which is our future work. Moreover, we will also implement the proposed Z-LDA to our online BCI system.
